# ORGANIZATIONAL SUPPORTS ASSOCIATED WITH NURSING ASSISTANTS’ JOB SATISFACTION DURING COVID-19

**DOI:** 10.1093/geroni/igac059.2569

**Published:** 2022-12-20

**Authors:** Natasha Bryant, Verena Cimarolli, Francesca Falzarano, Robyn Stone

**Affiliations:** LeadingAge, Washington, District of Columbia, United States; LeadingAge, Washington, District of Columbia, United States; Weill Cornell Medicine, Douglaston, New York, United States; LeadingAge, Washington, District of Columbia, United States

## Abstract

The COVID-19 pandemic has disproportionately affected nursing home residents and staff, including the nursing assistants who provide critical supports for older adults and people with disabilities. The pandemic has added significant strain to an already vulnerable nursing home workforce, which has historically experienced high levels of turnover, chronic staffing shortages, and high burnout. At the same time, it has generated awareness of the value of the direct care workforce to provide care in places for those most at risk from the disease. Job satisfaction of nursing assistants is as major driver of turnover and intent to leave the job. Research has demonstrated organizational supports and job stresses that are associated with job satisfaction and turnover. However, limited research has investigated the factors associated with job satisfaction in nursing homes during the COVID-19 pandemic. Using data from an employee engagement and management system, we examined organizational supports (e.g., supervision, appreciation, safety) and work-related stresses associated with job satisfaction among nursing assistants in nursing homes (n=402). Higher quality of supervision, feeling appreciated for the job, and feeling safe and comfortable at work were associated with higher levels of job satisfaction. Work-related job stressors –increased workload demands and understaffing – were associated with lower job satisfaction. Our findings provide insights into the importance of employer supports and reducing work-related stressors in nursing assistants’ job satisfaction and practical implications for nursing home leadership regarding how to support workers

